# Reconfigurable Multipoint Forming Using Waffle-Type Elastic Cushion and Variable Loading Profile

**DOI:** 10.3390/ma13204506

**Published:** 2020-10-12

**Authors:** Mohammed Moheen, Adel Abdel-Wahab, Hany Hassanin, Khamis Essa

**Affiliations:** 1 Department of Mechanical Engineering, School of Engineering, University of Birmingham, Edgbaston, Birmingham B15 2TT, UK; mxm580@students.bham.ac.uk (M.M.); a.a.m.abdelwahab@bham.ac.uk (A.A.-W.); 2 School of engineering, technology and design, Canterbury Christ Church University, Canterbury CT1 1QU, UK; enghanisalama@yahoo.com

**Keywords:** multi-point forming, finite-element modelling, response surface methodology, sheet metal forming, analysis of variance

## Abstract

There is an increasing demand for flexible, relatively inexpensive manufacturing techniques that can accommodate frequent changes to part design and production technologies, especially when limited batch sizes are required. Reconfigurable multi-point forming (MPF) is an advanced manufacturing technique which uses a reconfigurable die consisting of a set of moveable pins to shape sheet metal parts easily. This study investigates the use of a novel variable thickness waffle-type elastic cushion and a variable punch-loading profile to either eliminate or minimise defects associated with MPF, namely wrinkling, thickness variation, shape deviation, and dimpling. Finite element modelling (FEM), analysis of variance (ANOVA), and the response surface methodology (RSM) were used to investigate the effect of process parameters pertaining to the cushion dimensions and type of loading profile on the aforementioned defects. The results of this study indicate that the most significant process parameters were maximum cushion thickness, cushion cut-out base radius, and cushion cut-out profile radius. The type of loading profile was found to be insignificant in all responses, but further investigation is required as the rate, and the thermal effects were not considered in the material modelling. Optimal process parameters were found to be a maximum cushion thickness of 3.01 mm, cushion cut-out base radius of 2.37 mm, cushion cut-out profile radius of 10 mm, and a “linear” loading profile. This yielded 0.50 mm, 0.00515 mm, 0.425 mm for peak shape deviation, thickness variation, and wrinkling, respectively.

## 1. Introduction

Mass customisation is in continuous demand as a promising approach to combine personalization and flexibility of custom-produced parts with low mass-manufacturing costs. However, mass customisation conflicts with the current production lines, which produce identical parts with high quantities. Additive manufacturing is a technology to manufacture highly customised components but in small quantities. The technology has achieved rapid worldwide popularity because of its ability to manufacture parts with high geometric freedom and material utilisation. The technology enables the processing of a wide range of materials, including, polymers [1], ceramics [2,3,4,5,6], metals [7], and composites [8] which promotes the adoption of this technology in healthcare [9], defence [10], energy [11], and aerospace [12,13]. However, for large and curvilinear sheet-metal surfaces as in the automotive industry, additive manufacturing is not state of the art. The poor surface roughness, slow-building rate, especially with large parts, and the anisotropy properties of the fabricated parts along with the need for post-processing steps are undesirable in automotive sectors [14]. 

Traditional sheet metal manufacturing methods involve plastically deforming a metallic sheet using a set of complementary dies configured to a designated geometry. These methods are widely used in large-scale production to manufacture high-quality products quickly and inexpensively. However, due to the high tooling costs and time expense associated with traditional methods, they are suboptimal where limited batch sizes are required. In recent years, the requirement for flexible manufacturing processes which can accommodate frequent changes to the part design with minimal expense has risen drastically [15,16]. Multi-point forming (MPF) is one such process; it replaces its solid dies for an ordered set of discrete pins which can generally move to the workpiece to construct a pseudo-die surface [17]. 

MPF has seen considerable progress with regards to its viability as a manufacturing process, as well as in the removal of defects associated with it, namely dimpling, wrinkling, and springback [18]. Several investigations have studied the effect of pin tip and shape on surface quality [19,20,21]. Schuh et al. [21] recommended partial spherical pin tips based on machinability and formability but reported that hemi-ellipsoidal pin tips covered the largest range of contact angles. Walczyk and Hardt [22] recommended square-based pins as only they offered load path isolation. They also investigated other MPF design factors, including pin clamping and containment, forming force capacity, and die surface formation. Park et al. used a design of experiment approach to investigate the effect of process parameters on stress distribution, forming force, and spring back [23].

Paunoiu et al. [24] developed bespoke finite element (FE) models for MPF based on the pin contact points. They concluded that localised deformation is significant in MPF and is heavily dependent on contact points and, hence, recommended using an interpolator between the die and cushion to improve surface quality. Similarly, Zhang et al. [25] proposed a variation of the established MPF method, namely multi-point sandwich forming (MPSF) to reduce process defects. Gorgi et al. [26] investigated the effect of inhomogeneities on the plastic strain of metal parts. The results showed that the presence of inhomogeneities enables an accurate estimation of localized necking. In MPSF, the punch pin matrix is replaced by a deformable die and a polyurethane interpolator, and FEM was used to model the stress distribution and springback. They concluded that the presence of a pliable interpolator could produce smooth surface quality and reduce the amount of dimpling seen. Zhong-qin et al. [27] proposed an optimisation algorithm for blank holder force (BHF); their algorithm would vary the force with punch stroke. The variable BHF was found to improve the forming limit of the workpiece by circa 30%. Liu et al. [28] investigated the effect of a novel layered blank holder that deformed in tandem with the workpiece. They reported that the design eliminated wrinkling, improved stress and strain distribution homogeneity, and thickness variation across the workpiece. Qu et al. [29] implemented a segmented strip steel pad which was located between the dies and the elastic cushion. They analysed the impact to final part quality experimentally and with finite element modelling (FEM). In both cases, they observed that the presence of the steel pad increased friction force and generated a surface compressive stress on the workpiece. This reduced the wrinkling, dimpling, springback, and straight edge defects in the final part. Quan et al. [25] and Zareh-Desari et al. [30] investigated the effect of the elastic cushion, with the former also studying the impact of cushion thickness. They both found that the presence of the elastic cushion is necessary to minimise dimpling defects and improve forming accuracy. Cai et al. [27] used FEM to investigate wrinkling, dimpling, and springback. They found that wrinkling wave amplitude increases with the stroke of the punch until a critical point is reached whereby the amplitude decreases to a final value and then becomes invariant. Statistical modelling, namely analysis of variance (ANOVA) and design of experiments (DOE), is used extensively in tool and process design to analyse parameter significance and parameter interactions. Essa et al. optimised the process parameters using DOE for a sheet metal spinning process [31] and single point incremental forming [32]. Similarly, Majagi et al. employed a Box–Behnken design of experiments along with a response surface methodology to study several factors such as speed, feed rate, and coolant on the surface roughness, thickness reduction, and hardness of aluminium sheet [33]. Elgahwail et al. [34] employed the response surface DOE and the analysis of variance to identify the optimsed process parameters of MPF process on the amount of springback. The effect of coefficient of friction, pin size, cushion thickness, and radius of curvature response surface method in order to minimise process defects and improve final part quality. To the best of the author’s knowledge, although the effect of conventional and mesh-type elastic cushions have been investigated, no study currently exists for using a waffle-type cushion with a variable thickness profile. Furthermore, an investigation into different punch loading profiles has not yet been conducted. This work aims to employ FEM and the face-centred response surface method (RSM) to investigate the effect of maximum cushion thickness, cushion cut-out base radius, cushion cut-out profile radius, and punch loading profile on final part quality. The quality characteristics that will be considered are the thickness variation, peak shape deviation, and wrinkling.

## 2. Experimental and Methods 

### 2.1. Materials Properties

A steel sheet made of DC05 with a thickness of 1 mm was employed in this work. DC05 is popular non-alloy steel that is used for cold-forming techniques of complex shaped parts such as deep drawing and incremental forming. The composition of the material is shown in Table 1.

The mechanical properties of the DC05 blank sheet were obtained using universal mechanical tester, Zwick/Roell Z030, Leominster, UK. The sheet metal samples were cut according to ASTM E8 standard [35], and an extensometer was attached to the specimen. The properties of DC05 steel given in Table 2.

The material was assumed to be isotropic and homogeneous, and the elastic-plastic model was used. Flow stress was assumed to obey a reduced Hollomon power law:

(1) $$\sigma =K\varepsilon^{n}$$

where σ refers to true stress, n is the strain hardening exponent, ε is the true strain, and K is the strength coefficient.

**Figure 1:**
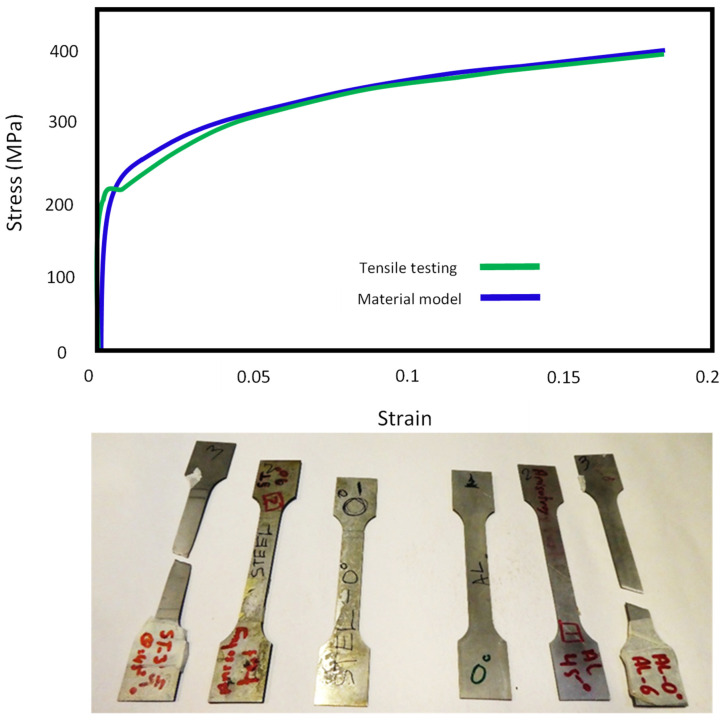
Material model and experimental stress strain curve of DC05 steel.

The parameters of the reduced Hollomon power law (n and K) were found by fitting Equation (1) to the stress-strain curve of DC05 sheet steel obtained by uniaxial tension test using universal mechanical tester, Z030 (Zwick/Roell, Leominster, UK). A comparison between the material model and the experimentally obtained tensile test properties is shown in Figure 1.

### 2.2. Numerical Modelling of Multi-Point Forming (MPF)

**Figure 2:**
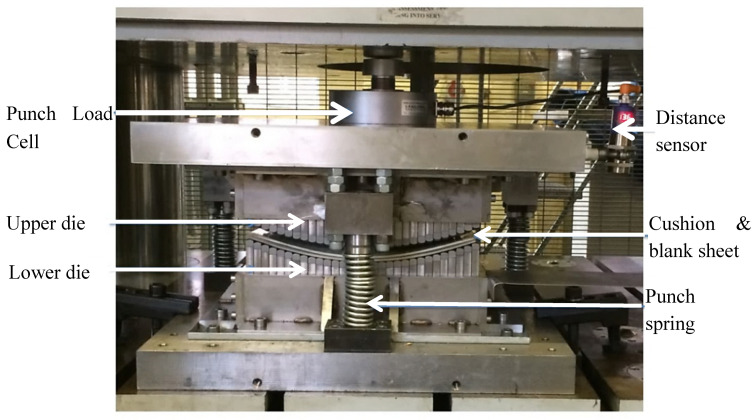
Reconfigurable multipoint forming setup.

A finite element model was developed based on the MPF tool in Figure 2 using ABAQUS CAE 2018 (Dassault Systèmes, Vélizy-Villacoublay, France). The setup is shown in Figure 2 consists of a set of 30 × 20 pin matrices, two elastic cushions, and a workpiece. To reduce the computational cost, only a quarter of the setup was simulated because of geometrical symmetry in the X and Z directions. The pins had general dimensions of 10 mm × 10 mm × 10 mm, with the pin tips possessing 10 mm spherical curvature. Pin separation was set at 0.25 mm. The workpiece was modelled as a DC05 steel sheet of dimensions 153.5 mm × 102.5 mm × 1 mm and was set to have a final geometry of 400 mm spherical curvature.

**Figure 3:**
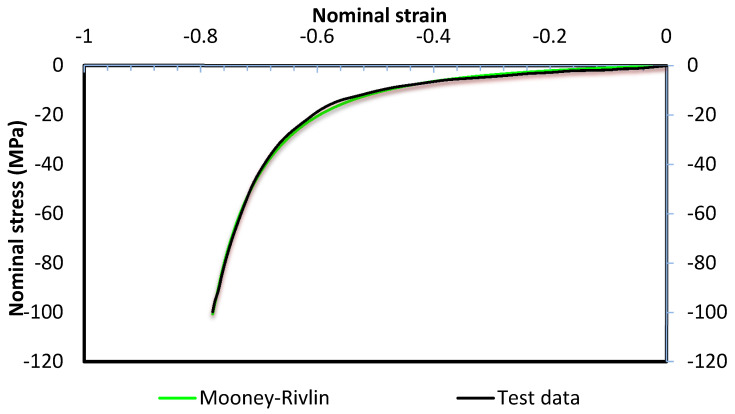
Compression testing diagram compared to Mooney–Rivlin model.

An elastic cushion is placed between the pin matrices and the workpiece. The general dimensions of the cushion used for model validation were 153.5 mm × 102.5 mm × 3 mm. The material of the cushion was Polyurethane–A90; it was treated as isotropic and had a density of 1130 kg/m^3^. The compression properties of polyurethane A–90 were carried out using universal mechanical tester, Zwick/Roell Z030, Leominster, UK. The compression results were compared with the Mooney–Rivlin model according to Equation (2). A good agreement was found between the two models, see Figure 3.

(2) $$W=C_{01}\left({\bar{I}}_{1}-3\right)+C_{10}\left({\bar{I}}_{2}-3\right)+\frac{1}{D_{1}}{\left(J-1\right)}^{2}$$

where *W* is the strain energy density, ${\bar{I}}_{1}$ and ${\bar{I}}_{2}$ are the first and second invariants of the deviatoric strain tensor, *J* is the elastic volume ratio for isotropic thermal expansion, $C_{01}$ and $C_{10}$ are coefficients relating to deviatoric response, and $D_{1}$ is a coefficient relating to the volumetric response obtained from a uniaxial compression test conducted using a Shore hardness of 90. The values of $C_{01}$ and $C_{10}$ are 0.861 and 0.354, respectively, Abosaf et al. [15].

The Poisson’s ratio of polyurethane–A90 is defined by Equation (3).

(3) $$\nu =\frac{3K_{0}/\mu_{0}-2}{6K_{0}/\mu_{0}+2}$$

where, $\;K_{0}$ and $\mu_{0}$ refer to the initial bulk and shear moduli of the material, respectively. The material was assumed to be incompressible (ν≅0.5 ) due to lack of material data with evidence to the contrary. However, due to numerical stability constraints, true incompressibility cannot be directly modelled in ABAQUS/Explicit. Thus it was assumed that the material was almost incompressible such that ${\left(J-1\right)}^{2}\approx 0$ and $\;K_{0}/\mu_{0}=20$. This corresponded to a Poisson’s ratio of 0.475.

**Figure 4:**
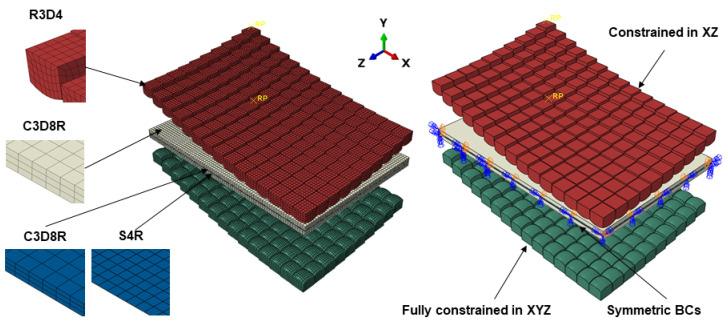
Schematic of the initial finite element (FE) model, including element type and boundary conditions (BCs).

An overview of the FE model is shown in Figure 4. A general contact algorithm was used to define interfacial contact. A friction coefficient of 0.1 was assumed between all bodies to model tangential behaviour, and this was achieved using penalty formulation [36,37,38].

Normal behaviour was not considered in the model. The pin matrices, namely the punch and die, were modelled as rigid bodies and were meshed using R3D4 elements. In order to validate the model against previous work from our research group, the workpiece and cushion were defined as deformable and were meshed using C3D8R elements [34,36,39]. Also, shell elements, S4R, were used to mesh the workpiece as their computational cost is small, and the thickness distribution could be gauged more easily. Mesh sensitivity analysis was conducted to identify the correct element size at which a solution can be reached in a reasonable time without the model being mesh dependent. The number of elements in the punch, die, and cushion were 30,900, 30,900, and 11,781, respectively. The workpiece consisted of 47,586 and 15,862 for continuum-solid and shell elements, respectively. Symmetric boundary conditions corresponding to the X and Z directions were applied to the workpiece and cushion, see Figure 4. A boundary condition was applied to the die to constrain it in all six-degrees-of-freedom (DOF). Similarly, a displacement boundary condition was used on the punch to constrain it in XYZ rotationally and XZ translationally, see Figure 4. Punch displacement was set at 42.2 mm in the Y direction, and an amplitude operator was used to vary the loading profile.

**Figure 5:**
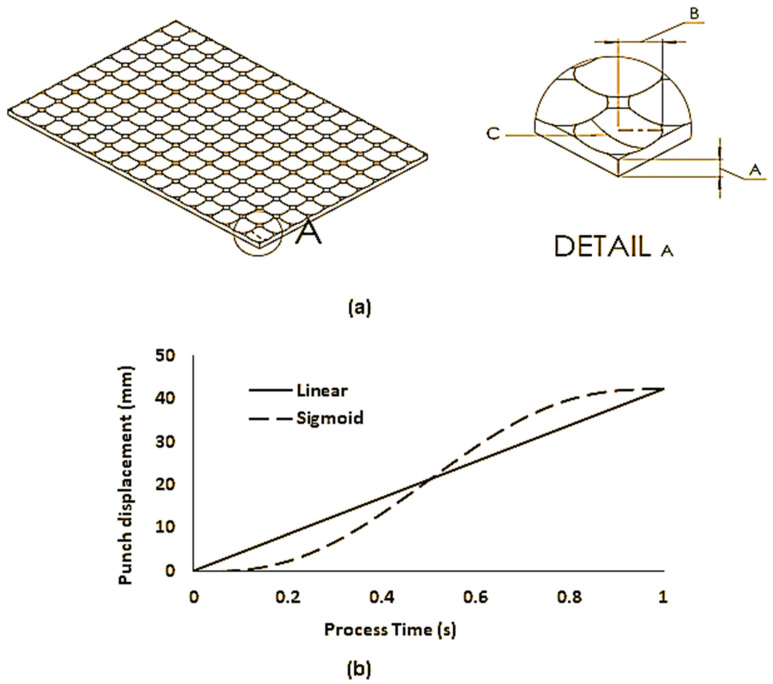
(**a**) Overall geometry of the waffle-type elastic cushion with a variable thickness profile. The dimensions varied in this study are A, B, and C. A is the maximum cushion thickness, B is the cushion cut-out base radius, C is the cushion cut-out profile radius; (**b**) linear and sigmoid punch loading profiles.

In this work, “sigmoid” and “linear” loading profiles were studied; these are shown in Figure 5 along with the overall shape and dimensions of the waffle-type cushion investigated. As can be seen in Figure 5, the waffle-type cushion consisted of a set of ordered curved cut-outs at locations directly typical to the pin travel paths. These cut-outs were defined using two main dimensions, the spherical curvature of the cut out (henceforth referred to as the cut-out profile radius) and the width of the cut-out (henceforth referred to as the cut-out base radius). As stated above, ABAQUS 2019 explicit software (Dassault Systèmes, Vélizy-Villacoublay, France) was employed; this was done to avoid convergence issues due to the non-linear deformation, a large number of elements, and the complex contact conditions involved in this problem. All simulations were performed on an Intel^®^ CoreTM i5-7300HQ processor (Intel Corporation, Mountain View, CA, USA) at 2.50 GHz. The analyses were performed using double precision to avoid round-off errors, and parallel processing was used due to the large node count. To reduce simulation time, a mass scaling factor of 10,000 was used for both the C3D8R and S4R element models; this reduced computation time significantly without sacrificing numerical accuracy.

### 2.3. Model Validation

**Figure 6:**
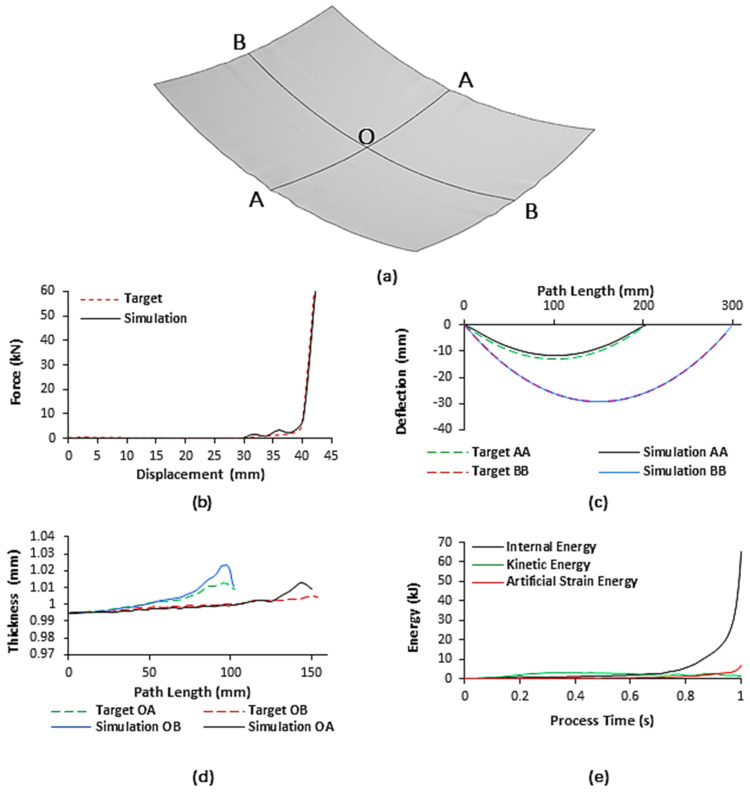
(**a**) Schematic overviewing principal axes of the deformed workpiece; (**b**) the force-displacement output of the C3D8R finite element (FE) model compared to the target profile; (**c**) deformed profile output of C3D8R FE model across principal axes compared to the target profile; (**d**) thickness distribution output of S4R FE model across principal axes to centroid compared to the target distribution; (**e**) energy history output of the C3D8R FE model.

The FE model was validated against experimentally published results obtained, in the same research group, by Abosaf et al. [39] where a flat cushion and linear punch loading profile were used. Figure 6 shows a comparison between the simulation results of the developed model and experimental results (target) obtained by Abosaf et al. [39]. Figure 6a shows the deformed workpiece and Figure 6 shows the comparison of the force-displacement, shape, and thickness distribution profiles to their respective targets, whereas Figure 6e shows the energy history output of the FE model. As shown in Figure 6b, it can be observed that the forming force increases gradually up until 40 mm displacement at which point, all pins were in direct contact with the elastic cushion, and plastic deformation has commenced. After this point, work-hardening of the material leads to a sharp rise to 60.1 kN until the end of motion [40]. This results in a percentage error of 2% when compared to the 58.9 kN target. For the shape profile, Figure 6c, the peak deflections observed occurred at the centre of the sheet and were found to be −11.82 mm and −29.14 mm for axes AA and BB, respectively. Comparing these simulation outputs to the targets of −13.17 mm and −29.35 mm yields percentage errors of 10.3% and 0.7%, respectively.

In the case of thickness distribution, Figure 6d, the workpiece is thinner nearer its centre and becomes thicker closer to the flange. This occurs as the centre of the sheet undergoes the most significant level of plastic deformation leading to sheet thinning and outward material flow. The peak thickness was found to be 1.013 mm and 1.023 mm for axes OA and OB, respectively. The relative change in values compared to the original workpiece thickness of 1 mm is very small, indicating that the normal to longitudinal plastic strain is approximately equal, which is ideal in sheet-forming processes. Comparing these outputs to the targets of 1.005 mm and 1.013 mm yields percentage errors of 0.8% and 1.0%, respectively. Overall, the developed model sees a good agreement with the experimental results obtained by Abosaf et al. [39] with a maximum error of 10.3%. To ensure reliability, it was necessary to check the stability of the solution given by the FE model. This was achieved by confirming that the workpiece deforms quasi-statically and element distortion via hourglassing is kept to a minimum. From Figure 6e, the kinetic energy (KE) and artificial strain energy (AE) at the end of the analysis total 2.1% and 10.2% of the internal energy (IE). As the KE is less than 5% of the IE, then, inertial forces can be considered small enough to not dominate the solution [41]. For the FE model to be reliable, the maximum KE of the deformed material and the maximum AE must both be less than 10% of the maximum IE [42]. As the KE is 2.1% of the IE and the AE is approximately 10% of the IE [43], it was sufficient to conclude that artificial deformation had minimal impact on the solution and the FE model can be considered reliable.

### 2.4. Statistical Validation

Statistical methods, such as DOEs and ANOVA, have been used widely in manufacturing to investigate, predict, and optimise process response to a change in process parameters. A face-centred RSM was used to generate a set of experiments for analysing the effects of maximum cushion thickness, cut-out base radius, cut-out profile radius, and the type of punch-loading profile on the defects seen in MPF. First-order orthogonal response-surface methods are generally used over a narrow set of process parameters; hence a second-order polynomial model was selected. This is given by the general expression [14]:

(4) $$R=\beta_{0}+\overset{n}{\underset{l=1}{\sum }}\beta_{l}x_{m}+\overset{n}{\underset{l=1}{\sum }}\beta_{ll}x_{l}^{2}+\sum \underset{l<m}{\sum }\beta_{lm}x_{l}x_{m}+\epsilon$$

where R is the process response, x is any of the studied process factors, β refers to the polynomial coefficients, and ϵ refers to the random error. The coefficients were derived using non–linear least–squares analysis. In this work, for each continuous parameter, three levels were tested: −1, 0, and 1. As the punch-loading profile is a categoric factor, all continuous factor experiments were repeated for each level of that categoric factor, in this case, only two levels were tested. Table 3 summarises the process parameters (and their levels) which were used in the simulations.

The response variables correspond to the quality characteristics (QCs) of the final part [31]. In this work, only the quantitative QCs: thickness variation, wrinkling, and peak shape deviation, were considered in the DOE. Dimpling was considered a qualitative QC and was noted as being present when a non-uniform material distribution with highly localized strain was noticeably visible in the formed part. Wrinkling was defined as the normal deviation of the formed part from the target shape seen in Figure 6c when measured at the sheet flange. It was quantified as a root-mean-square-error (RSME) using Equation (5), *Z*_i_ is a single deviation of the formed part from the target shape [39]. To observe the trend more easily, only the wrinkling in the long edge of the deformed workpiece was used in the calculations.

(5) $$RMSE=\sqrt{\overset{n}{\underset{i=1}{\sum }}\frac{Z_{i}^{2}}{n}}$$

The sheet metal thickness was measured along the principal axes, and at the workpiece flanges, this was then quantified as a standard deviation, s (Equation (6) [29]), which was termed the thickness variation. Here, N denotes the number of points where the thickness was recorded, $x_{i}$ is a single data point, and $\bar{x}$ is the mean thickness in the data set.

(6) $$s=\sqrt{\overset{n}{\underset{i=1}{\sum }}\frac{{\left(x_{i}-\bar{x}\right)}^{2}}{N}}$$

Peak shape deviation was defined as the maximum normal distance between the target and formed part shapes, as stated previously, this would occur at the centre of the workpiece.

## 3. Results and Discussion

Table 4 shows the generated plan of 40 runs based on the DOE and the evaluated response of each QC in these simulations.

The simulation results were then analysed using Design Expert 12, and an ANOVA study was conducted to identify statistically significant parameters. In this investigation, both peak shape deviation and wrinkling were fitted using standard response modelling, whereas thickness variation required a logarithmic Box–Cox transformation. The coefficient of determination, R2, was found to be 91.66%, 90.46%, and 97.50% for peak shape deviation, thickness variation, and wrinkling, respectively. Similarly, the adjusted R2 values were found to be 87.49%, 85.68%, and 96.25% and the residuals were all approximately normally distributed, indicating good agreement with the quadratic model. 

A significance threshold of 5% was used for all parameters and parameters interactions. This assumed that parameters with *p*-values of less than 0.05 were deemed to be statistically significant. The smaller the p-value below this threshold, the more significant the process parameter [38,44]. The null hypothesis specified that none of the investigated parameters was significant.

Table 5 summarises the *p*-values of both the parameters tested and the two-factor interactions. The ANOVA results demonstrate that the maximum cushion thickness, cut-out base radius, and cut-out profile radius all have a significant impact on the peak shape deviation, thickness variation and wrinkling either as linear or quadratic terms.

In terms of importance, maximum cushion thickness is the most significant, followed by cut-out base radius and then by cut-out profile radius. Interestingly, the loading profile was deemed an insignificant parameter. One likely explanation is that due to the quasi-static nature of sheet forming, the change in the already low strain-rate between a linear profile and a sigmoid profile is too small to see a significant change in the measured response. However, it is difficult to conclude that the type of loading profile can be discounted as completely insignificant as the Hollomon material model that was used did not include rate and thermal effects. Hence, the impact to flow stress and measured strain between the two loading profiles is purely due to the change in punch speed which would be of small consequence in a quasi-static process. Further to this, the literature finds that the strain rate sensitivity, m, for DC05 steel is not insignificant, varying from 0.023 at low rates to 0.130 at high rates [45,46]. This argument is also supported by the fact that the *p*-values for the interactions between the type of loading profile and cut-out base radius in thickness variation and wrinkling are very close to the 0.05 threshold and thus would likely become significant if the aforementioned effects were included. Hence, further investigation into the deformation behaviour of DC05 steel in MPF under various strain-rates is likely warranted.

### 3.1. Peak Shape Deviation

**Figure 7:**
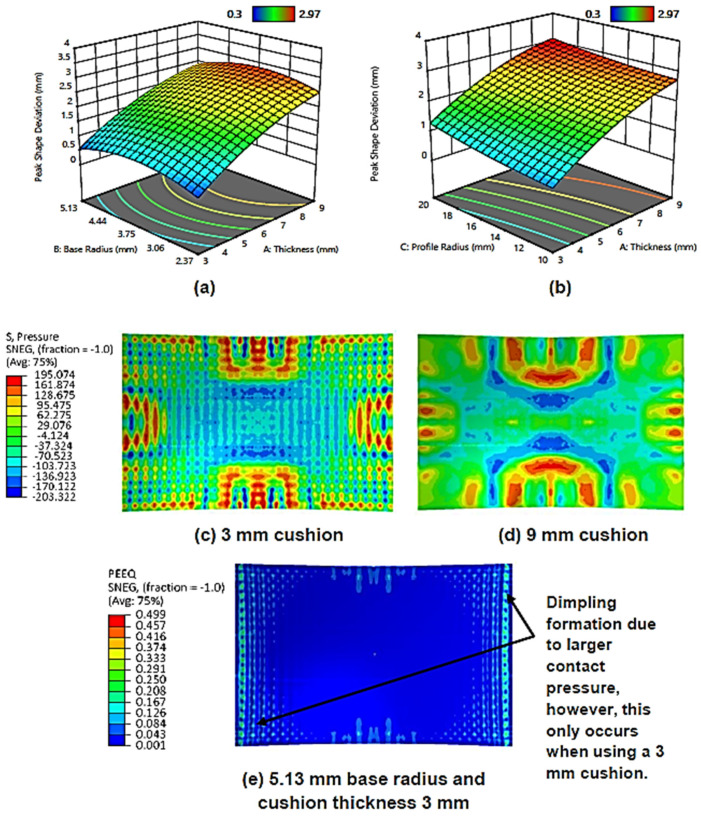
(**a**) Surface plot for the effect of maximum cushion thickness and cut-out base radius on peak shape deviation; (**b**) surface plot for the effect of maximum cushion thickness and cut-out profile radius on peak shape deviation; (**c**) pressure distribution contour (S4R) on the upper workpiece surface when using a 3 mm cushion; (**d**) pressure distribution contour (S4R) on the upper workpiece surface when using a 9 mm cushion; (**e**) equivalent strain contour (S4R) on upper workpiece surface when using a 3 mm cushion with 5.13 mm cut-out base radius and 10 mm cut-out profile radius.

Figure 7a shows the surface plot for the effect of maximum cushion thickness and cut-out base radius on the peak shape deviation. It can be observed that as the maximum cushion thickness increases, so too does the peak shape deviation. This is expected and is consistent with similar studies [34,38]. Increasing cushion thickness reduces local deformation and surface indentation. The larger material volume means that it can attenuate punch impact energy more effectively. This results in a reduced, more homogeneous pressure distribution as can be seen in Figure 7d, and an overall reduction in local sheet thinning. However, the lower stresses have the added drawback of leading to under-deformation of the workpiece and thus increased shape deviation [15,38]. A minimum error from the ideal shape is achieved when the maximum cushion thickness is 3 mm.

Although cut-out base radius is not statistically significant as a linear factor in peak shape deviation, it is as a quadratic one. It can be observed that the deviation is low when using base radii of 2.37 mm and 5.13 mm and is at its highest when an intermediate radius of 3.75 mm is used. At small radii, the variation in thickness across the cut-out is small, and so deformation behaviour is similar to that of a flat cushion. When the cushion is compressed, the surface adjacent to the pin matrices deforms, leading to a series of depressions at the point of contact [25]. Flatter cushions will exhibit smoother deformation during this process as their geometry is simple. As the base radius increases, cushion material flow in these regions becomes increasingly more complex, which may lead to non-uniform deformation of the workpiece and greater deviation. However, it is also true that increasing the base radius reduces the local cushion thickness, which acts to increase the transmitted punch contact pressure. This, in effect, will lead to better deformation and a smaller deviation. Thus, a potential explanation for the trend observed in Figure 7a, is that below 3.75 mm radius, the former effect is dominant, and beyond this point is when the latter phenomenon becomes dominant. If the cushion is thin; however, the latter phenomenon can also lead to the formation of the dimpling defect, this can be observed from the discontinuous, highly localised equivalent strain regions seen in Figure 7e.

Figure 7b shows the surface plot for cushion thickness and cut-out profile radius on peak shape deviation. It can be observed that as the profile radius decreases so too does the peak shape deviation with the minimum error being achieved at 10 mm curvature. One explanation for this trend pertains how the profile radius affects the contact conditions between the pins and the cushion.

**Figure 8:**
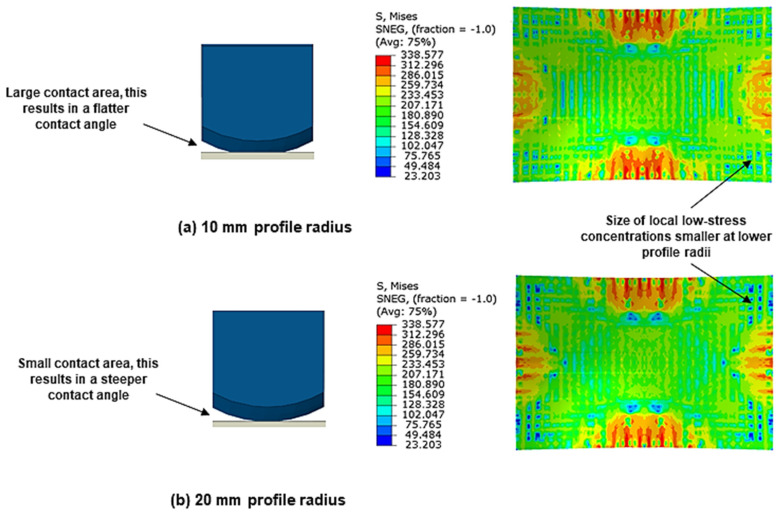
Comparison of the pin-to-cushion contact points and equivalent stress contours (S4R) on the upper workpiece surface when using (**a**) 10 mm profile radius and (**b**) 20 mm profile radius.

Figure 8 shows the effect of using small and large profile radii on pin-to-cushion contact and the developed stress distribution. It can be observed that as the profile curvature decreases, the pin-to-cushion contact area is increased, with the maximum area being achieved when the pin curvature matches that of the cut-out. This achieves a similar effect to increasing cushion thickness, albeit to a much smaller degree, whereby the pressure distribution becomes more even. This is seen in the equivalent stress contours where the size of local low-stress concentrations decreases at smaller profile radii, with this effect being most observable at the sheet corners. These low-stress regions will elastically recover whilst the surrounding material, which is plastically deforming, will not, causing uneven workpiece deformation to take place. Hence, the reduction in the size of these concentrations means that the stress is more uniform and local deformation is reduced. Additionally, decreasing the profile radius will not suppress macro–scale deformation of the entire workpiece as in the case of increasing maximum cushion thickness as there is no significant loss in the overall stress. Thus the overall deformation improves, thereby reducing deviation.

### 3.2. Sheet Metal Thickness Variation

**Figure 9:**
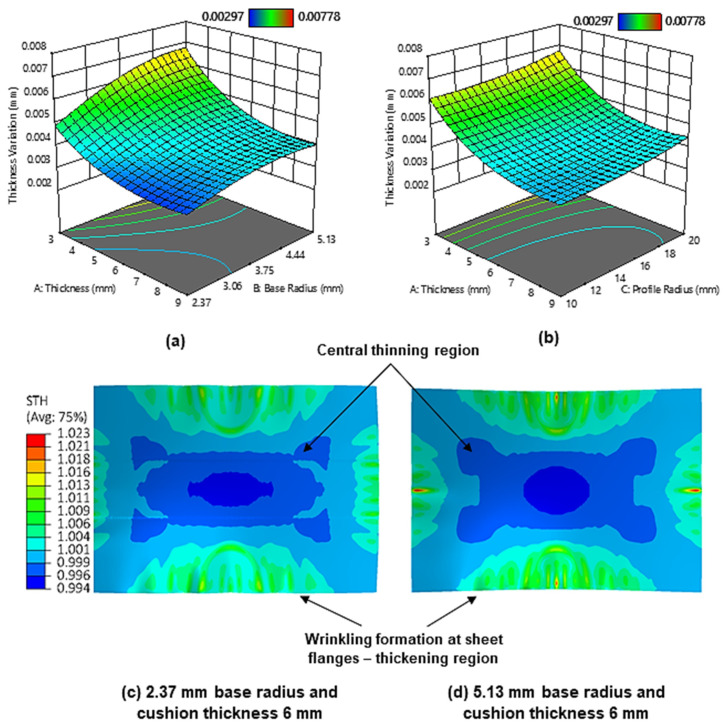
(**a**) Surface plot for the effect of maximum cushion thickness and cut-out base radius on sheet metal thickness variation; (**b**) surface plot for the effect of maximum cushion thickness and cut-out profile radius on sheet metal thickness variation; (**c**) section thickness contour on the upper workpiece surface when using a cut-out base radius of 2.37 mm; (**d**) section thickness contour on the upper workpiece surface when using a cut-out base radius of 5.13 mm.

Figure 9a shows the surface plot for the effect of cushion thickness and cut-out base radius on the thickness variation. It can be observed that as the maximum cushion thickness increases the sheet metal thickness variation decreases, with the minimum variation being achieved at 9 mm cushion thickness. As mentioned, thicker cushions lead to under-deformation; it follows that this results in reduced material flow outward from the workpiece centre, meaning the thickness across the sheet is more uniform overall. It should be noted that this differs from some existing results in the literature [34]. However, these can be attributed to some numerical modelling differences such as friction, and that these studies limited measuring thickness to only the principal axes of the workpiece, whilst in this work thickness at the workpiece flange was also considered. Moreover, the result found in this work is consistent with the results obtained by Abosaf et al. [15], so it can be considered to be reliable.

It is also observed that the sheet metal thickness variation increases as the cut-out base radius increases, with minimum thickness variation being achieved when a base radius of 2.37 mm is used. Figure 9c–d shows the thickness distribution contours when using small and large base radii. It can be observed that a larger base radius results in a larger sheet-thinning region. This is attributed to the aforementioned larger contact pressures at larger base radii generating more sheet stretching. Figure 9b shows the surface plot for the effect of cushion thickness and cut-out profile radius on the sheet metal thickness variation. As can be seen, the thickness variation decreases with decreasing profile radii curvature, with the minimum thickness variation being achieved at a 10 mm profile radius. As stated earlier, the smaller contact area and less uniform stress distribution at larger profile radii promote an increase in local deformation and cause the workpiece to deform more unevenly in regions where there are large differences in stress. This naturally leads to a more non-uniform workpiece thickness distribution as regions of high stress will exhibit more thinning than those of low stress.

### 3.3. Flange Wrinkling

**Figure 10:**
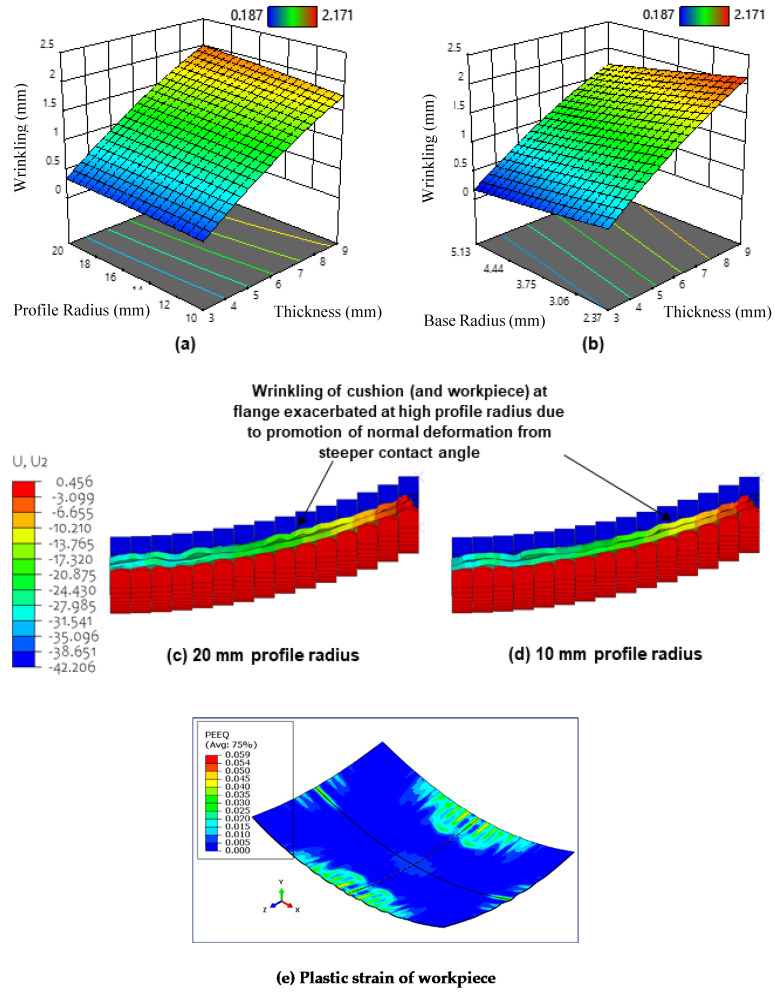
(**a**) Surface plot for the effect of maximum cushion thickness and cut-out profile radius on wrinkling; (**b**) surface plot for the effect of maximum cushion thickness and cut-out base radius on wrinkling; (**c**) displacement contour (S4R) of deformed workpiece and cushion (at the flange) when using a cut-out base radius of 20 mm; (**d**) displacement contour (S4R) of deformed workpiece and cushion (at the flange) when using a cut-out base radius of 10 mm; (**e**) plastic strain of the workpiece when two contact pins are used.

Wrinkling arises when in-plane tensile forces are insufficient, this can generate out-of-plane deformation in the form of wave-like perturbations. These are due to local plastic deformation that occur when some of the pins in the upper and lower dies start to establish contact with the sheet during the deformation process. The force starts to increase rapidly when all pins establish contact with the sheet until the maximum plastic deformation is reached. Figure 10a shows the surface plot for the effect of maximum cushion thickness and cut-out profile radius on wrinkling. It can be seen that wrinkling increases with cushion thickness, with maximum wrinkling being obtained at a thickness of 9 mm. This result agrees with findings in the literature [15].

According to Abebe et al. [47], the punch contact pressure has to exceed the induced compressive instabilities that are generated during deformation to eliminate wrinkles. When a thick cushion expands due to compression, a greater volume of material is forced to accumulate near the workpiece flange [48], this means that contact pressure in this region is reduced more so than in thinner cushions, it follows that fewer in-plane compressive instabilities are suppressed and wrinkle wave amplitude increases as a result. Figure 10b shows the surface plot for the effect of cushion thickness and cut-out base radius on wrinkling. It can be observed that wrinkling increases with decreasing cut-out base radius. This operates in the same manner as above, where the local reduction in cushion thickness and resulting increased pressure at larger base radii provide the necessary in-plane tensile force to counteract wrinkle formation. This is seen in Figure 8b, where the fraction of the workpiece that corresponds to the sheet thickening region decreases in size when the base radius is increased from 2.37 mm to 5.13 mm. 

It can also be seen from Figure 10a that wrinkling increases with increasing cut-out profile radius, with maximum wrinkling being obtained at 20 mm curvature. A possible explanation for this phenomenon is that the steeper contact angles at larger profile radii cause cushion material to flow in the normal direction when the cushion is compressed during the punch stroke. At the flange, this can lead to larger undulations and cause the cushion to depart slightly from the workpiece. This reduces the tangential tensile force that is exerted by the cushion (due to friction) on the workpiece in this region and promotes material to flow out-of-plane to fill the resulting departure regions inevitably exacerbating wrinkling defects [49,50]. This explanation is further supported when observing the deformed displacement contours shown in Figure 10c–d, where cushion wrinkling (and hence workpiece wrinkling) at the flange is seen to be relatively larger when using a 20 mm radius compared to 10 mm radius. It is also observed that the increase in wrinkling is rather small, especially when compared to the change in wrinkling observed when changing the maximum cushion thickness and cut-out base radius. This is because although more material does flow in the normal direction at larger profile radii, the magnitude of material flow in the lateral direction is still much larger in comparison. Figure 10e shows the plastic strain of the workpiece when two contact pins are used.

### 3.4. Optimisation of Process Parameters

Applying Equation (4) to the studied process parameters gives the general form of the governing equation for each response. This can be defined such that:

(7) $$R=\beta_{0}+\beta_{1}A+\beta_{2}B+\beta_{3}C+\beta_{4}D+\beta_{5}AB+\beta_{6}AC+\beta_{7}AD+\beta_{8}BC+\beta_{9}BD+\;\beta_{10}CD+\beta_{11}A^{2}+\beta_{12}B^{2}+\beta_{13}C^{2}+\beta_{14}D^{2}$$

 R refers to each QC or response variable; in the case of thickness variation, due to the logarithmic transformation, this is defined:

(8) $$R=\log_{10}s$$

where *s* is the thickness variation. A−D refer to the coded normalised values of maximum cushion thickness, cut-out base radius, cut–out profile radius, and punch loading profile, respectively. These are found using Equation (9):

(9) $$\mathrm{Coded}\;\mathrm{Normalised}\;\mathrm{Value}=\frac{2\left(\mathrm{Actual}\;\mathrm{Value}-\mathrm{Mean}\;\mathrm{Value}\;\mathrm{of}\;\mathrm{Range}\right)}{\mathrm{Highest}\;\mathrm{Value}-\mathrm{Smallest}\;\mathrm{Value}}$$

In the case of punch loading profile, “linear” was assigned a normalised value of −1 and “sigmoid” was assigned a normalised coded value of +1. Table 6 shows the values of the polynomial coefficients $\beta_{0}-\beta_{14}$.

Optimal parameters for minimising the studied responses were found using numerical optimisation, these are shown in Table 7. These optimal values were then validated using the same FE model. Table 8 compares the predicted responses, within a 95% confidence interval (± 1.96 standard deviation (S.D.)), to the measured responses. It can be observed that the predicted values are underestimated in the case of peak shape deviation and thickness variation and overestimated in the case of wrinkling. However, all the measured values fall within the 95% confidence interval of their predictions, this seems reasonable given the complex deformation behaviour associated with a variable thickness waffle-type elastic cushion.

## 4. Conclusions

In this study, FEM was used in tandem with RSM and ANOVA to investigate the deformation of a DC05 workpiece when using a variable thickness waffle-type elastic cushion and variable punch-loading profile in MPF. This study demonstrated the following:

1. The maximum cushion thickness, cut-out base radius, and cut-out profile radius were all significant process parameters in their effects on peak shape deviation, wrinkling, and thickness variation. In terms of importance, maximum cushion thickness was the most significant followed by cut-out base radius and then by cut-out profile radius. The quadratic model was found to be the best fit for the response variables investigated.
2. The type of punch-loading profile was deemed seemingly insignificant in all cases, but it is too early to completely discount it as rate and thermal effects were not considered in the FE model, thus further investigation is required.
3. Two-way interactions between process parameters were insignificant in all cases.
4. Maximum shape deviation was found to decrease with decreasing maximum cushion thickness and cut-out profile radius. For increasing the cut-out base radius, it was found to first increase then decrease.
5. Thickness variation was found to decrease with increasing maximum cushion thickness, decreasing cut-out base radius, and decreasing cut-out profile radius.
6. Wrinkling was found to decrease with decreasing maximum cushion thickness, decreasing cut-out profile radius, and increasing cut-out base radius.
7. In all cases, the results indicate that a waffle-type elastic cushion can be used to minimise the defects associated with MPF with optimal process parameters being found. However, further experimental investigations are still required.

## Figures and Tables

**Table 1:** Chemical composition of DC05 steel, as supplied.

Element	Mn	C	P	S	Fe
%	0.35	0.06	0.03	0.03	Balance

**Table 2:** Mechanical properties of DC05 steel.

DC05 Steel—Property	Value
Young’s Modulus, E	220 GPa
Density, ρ	7870 kg/m^3^
Yield Stress, $\sigma_{Y}$	200.6 MPa
Poisson’s ratio, ν	0.3
Fracture Strain, $\varepsilon_{F}$	0.181
Strength Coefficient, K	527.13 MPa
Hardening Exponent, n	0.17

**Table 3:** Process parameters and their corresponding levels.

Parameter	Unit	Level		
		−1	0	1
Maximum Cushion Thickness	mm	3.00	6.00	9.00
Cut–out Base Radius	mm	2.37	3.75	5.13
Cut–out Profile Radius	mm	10	15	20
Punch Loading Profile	-	-	Linear/Sigmoid	-

**Table 4:** Design of experiment (DOE) results for shape deviation, thickness variation, wrinkling, and dimpling as obtained when using S4R elements.

Std	Run	Max. Cushion Thickness (mm)	Cut-out Profile Radius (mm)	Cut-out Base Radius (mm)	Punch Loading Profile (–)	Peak Shape Deviation (mm)	Thickness Variation (mm)	Wrinkling (mm)	Dimpling (-)
13	1	6	3.75	10	Linear	2.19	0.00446	1.159	No
20	2	6	3.75	15	Linear	2.17	0.00431	1.159	No
5	3	3	2.37	20	Linear	1.06	0.00522	0.497	No
23	4	3	5.13	10	Sigmoid	0.45	0.00612	0.373	Yes
14	5	6	3.75	20	Linear	2.38	0.00511	1.353	No
27	6	3	5.13	20	Sigmoid	0.81	0.00695	0.251	No
29	7	3	3.75	15	Sigmoid	0.91	0.00778	0.276	No
21	8	3	2.37	10	Sigmoid	0.50	0.00546	0.372	No
38	9	6	3.75	15	Sigmoid	2.22	0.00417	1.283	No
1	10	3	2.37	10	Linear	0.30	0.00495	0.426	No
33	11	6	3.75	10	Sigmoid	2.22	0.00412	1.218	No
39	12	6	3.75	15	Sigmoid	2.22	0.00417	1.283	No
25	13	3	2.37	20	Sigmoid	1.20	0.00555	0.652	No
35	14	6	3.75	15	Sigmoid	2.22	0.00417	1.283	No
7	15	3	5.13	20	Linear	0.88	0.00699	0.299	No
6	16	9	2.37	20	Linear	2.97	0.00395	2.031	No
9	17	3	3.75	15	Linear	1.07	0.00574	0.334	No
30	18	9	3.75	15	Sigmoid	2.74	0.00422	1.922	No
15	19	6	3.75	15	Linear	2.17	0.00431	1.159	No
11	20	6	2.37	15	Linear	0.88	0.00297	1.573	No
22	21	9	2.37	10	Sigmoid	2.24	0.00335	1.818	No
31	22	6	2.37	15	Sigmoid	2.11	0.00391	1.415	No
26	23	9	2.37	20	Sigmoid	2.62	0.00396	2.005	No
36	24	6	3.75	15	Sigmoid	2.22	0.00417	1.283	No
19	25	6	3.75	15	Linear	2.17	0.00431	1.159	No
37	26	6	3.75	15	Sigmoid	2.22	0.00417	1.283	No
12	27	6	5.13	15	Linear	2.16	0.00536	1.012	No
16	28	6	3.75	15	Linear	2.17	0.00431	1.159	No
40	29	6	3.75	15	Sigmoid	2.22	0.00417	1.283	No
34	30	6	3.75	20	Sigmoid	2.33	0.00466	1.315	No
18	31	6	3.75	15	Linear	2.17	0.00431	1.159	No
4	32	9	5.13	10	Linear	1.89	0.00353	1.414	No
8	33	9	5.13	20	Linear	2.60	0.00410	1.909	No
17	34	6	3.75	15	Linear	2.17	0.00431	1.159	No
32	35	6	5.13	15	Sigmoid	2.16	0.00438	1.135	No
10	36	9	3.75	15	Linear	2.94	0.00390	2.102	No
3	37	3	5.13	10	Linear	0.34	0.00645	0.187	Yes
2	38	9	2.37	10	Linear	2.84	0.00320	2.171	No
28	39	9	5.13	20	Sigmoid	2.56	0.00409	2.012	No
24	40	9	5.13	10	Sigmoid	2.30	0.00397	1.505	No

**Table 5:** Significance of the investigated process parameters and any two-factor interactions. Values highlighted in bold indicate that the *p*-values fall below the significance threshold of 5%.

Process Parameter	Units	Response Factors					
		Peak Shape Deviation		Thickness Variation		Wrinkling	
Cushion Thickness (A)	mm	< 0.0001		< 0.0001		< 0.0001	
Cut-out Base Radius (B)	mm	0.6395		< 0.0001		< 0.0001	
Cut-out Profile Radius (C)	mm	0.0020		0.0049		0.0022	
Punch Loading Profile (D)	-	0.5813		0.4919		0.4418	
Quadratic Terms	-	A^2^ = 0.0169 B^2^ = 0.0027 C^2^ = 0.5341		A^2^ ≤ 0.0001 B^2^ = 0.0127 C^2^ = 0.4261		A^2^ = 0.0230 B^2^ = 0.8117 C^2^ = 0.8147	
Two-Factor Interactions	-	AB = 0.4976 AC = 0.4208 AD = 0.4133	BC = 0.9267 BD = 0.8627 CD = 0.6991	AB = 0.0913 AC = 0.3867 AD = 0.6508	BC = 0.8513 BD = 0.0895 CD = 0.6447	AB = 0.4383 AC = 0.1214 AD = 0.2795	BC = 0.1907 BD = 0.0831 CD = 0.6643

**Table 6:** Polynomial coefficients for response variable equations.

Polynomial Coefficient	Response Variable		
	Peak Shape Deviation (mm)	Thickness Variation (log_10_(mm))	Wrinkling (mm)
$\beta_{0}$	2.20	−2.36	1.24
$\beta_{1}$	0.9090	−0.1007	0.7611
$\beta_{2}$	−0.0285	0.0421	−0.1432
$\beta_{3}$	0.2070	0.0237	0.0840
$\beta_{4}$	0.0238	0.0038	0.0136
$\beta_{5}$	−0.0463	−0.0152	−0.0218
$\beta_{6}$	−0.0550	0.0076	0.0443
$\beta_{7}$	0.0500	−0.0035	−0.0273
$\beta_{8}$	−0.0063	−0.0016	0.0371
$\beta_{9}$	−0.0105	−0.0136	0.0446
$\beta_{10}$	−0.0260	−0.0036	0.0109
$\beta_{11}$	−0.2927	0.0682	−0.1139
$\beta_{12}$	−0.3802	−0.0395	0.0113
$\beta_{13}$	0.0723	0.0119	−0.0112
$\beta_{14}$	0	0	0

**Table 7:** Optimal conditions for minimal defects.

Condition	Max. Cushion Thickness (mm)	Cut-out Base Radius (mm)	Cut-out Profile Radius (mm)	Loading Profile (-)
Optimal	3.01	2.37	10	Linear

**Table 8:** Comparison of predicted response variables from the optimal setting and the measured values.

Results	Peak Shape Deviation (mm)	Thickness Variation (mm)	Wrinkling (mm)
Predicted	0.30 ± 1.96 (0.27)	0.00475 ± 1.96 (0.00038)	0.503 ± 1.96 (0.111)
Measured	0.50	0.00515	0.425

## Nomenclature

MPF	Multi-point forming
IE	Internal Energy
SE	Strain Energy
DOE	Design of Experiments
RSM	Response Surface Methodology
ANOVA	Analysis of Variance
FEM	Finite Element Modelling
BHF	Blank Holder Force
DOF	Degrees of Freedom
E	Young’s Modulus
σ	True Stress
ε	True Strain
k	Coefficient of Strength
n	Strain Hardening Exponent
ν	Poisson’s Ratio
ρ	Density
$\sigma_{Y}$	Yield Strength
$\sigma_{F}$	Fracture Strength
W	Strain Energy Density
$C_{01}/C_{10}$	Deviatoric Response Coefficient
$D_{1}$	Volumetric Response Coefficient
$\bar{I_{1}}/\bar{I_{2}}$	Invariants of Deviatoric Strain Tensor
$J_{1}$	Elastic-volume Ratio for Thermal Expansion
$K_{0}$	Initial Bulk Modulus
$\mu_{0}$	Initial Shear Modulus
R	Response Variable
x	Process Parameter
*β*_0_ – *β*_14_	Polynomial Response Coefficients
ϵ	Random Process Error
QC	Quality Characteristic
RSME	Root Mean Square Error
$Z_{i}$	Amplitude of Wrinkle Wave
n	Number of Wrinkling Waves
s	Thickness Variation Given as a Standard Deviation
$x_{i}$	Data Point
$\bar{x}$	Mean Thickness of Data Set
N	Number of Points in Data Set
A	Normalised Maximum Cushion Thickness
B	Normalised Cushion Cut-out Base Radius
C	Normalised Cushion Cut-out Profile Radius
D	Normalised Punch Loading Profile
SD	Forecast Standard Deviation
